# Influence of Interspecies Transmission of Atypical Bovine Spongiform Encephalopathy Prions to Hamsters on Prion Characteristics

**DOI:** 10.3389/fvets.2020.00094

**Published:** 2020-03-03

**Authors:** Kohtaro Miyazawa, Kentaro Masujin, Yuichi Matsuura, Yoshifumi Iwamaru, Hiroyuki Okada

**Affiliations:** ^1^Viral Ecology Unit, National Institute of Institute of Animal Health (NIAH), National Agriculture and Food Research Organization (NARO), Tsukuba, Japan; ^2^African Swine Fever Unit, NIAH, NARO, Tokyo, Japan; ^3^Department of Planning and General Administration, NIAH, NARO, Tsukuba, Japan

**Keywords:** atypical bovine spongiform encephalopathy, H-BSE, L-BSE, interspecies transmission, prion strain, hamsters

## Abstract

Bovine spongiform encephalopathy (BSE) is a prion disease in cattle and is classified into the classical type (C-BSE) and two atypical BSEs, designated as high type (H-BSE) and low type (L-BSE). These classifications are based on the electrophoretic migration of the proteinase K-resistant core (PrP^res^) of the disease-associated form of the prion protein (PrP^d^). In a previous study, we succeeded in transmitting the H-BSE prion from cattle to TgHaNSE mice overexpressing normal hamster cellular PrP (PrP^C^). Further, Western blot analysis demonstrated that PrP^res^ banding patterns of the H-BSE prion were indistinguishable from those of the C-BSE prion in TgHaNSE mice. In addition, similar PrP^res^ glycoprofiles were detected among H-, C-, and L-BSE prions in TgHaNSE mice. Therefore, to better understand atypical BSE prions after interspecies transmission, H-BSE prion transmission from TgHaNSE mice to hamsters was investigated, and the characteristics of classical and atypical BSE prions among hamsters, wild-type mice, and mice overexpressing bovine PrP^C^ (TgBoPrP) were compared in this study using biochemical and neuropathological methods. Identical PrP^res^ banding patterns were confirmed between TgHaNSE mice and hamsters in the case of all three BSE prion strains. However, these PrP^res^ banding patterns differed from those of TgBoPrP and wild-type mice infected with the H-BSE prion. In addition, glycoprofiles of TgHaNSE mice and hamsters infected with the L-BSE prion differed from those of TgBoPrP mice infected with the L-BSE prion. These data indicate that the PrP^C^ amino acid sequences of new host species rather than other host environmental factors may affect some molecular aspects of atypical BSE prions. Although three BSE prion strains were distinguishable based on the neuropathological features in hamsters, interspecies transmission modified some molecular properties of atypical BSE prions, and these properties were indistinguishable from those of C-BSE prions in hamsters. Taken together, PrP^res^ banding patterns and glycoprofiles are considered to be key factors for BSE strain typing. However, this study also revealed that interspecies transmission could sometimes influence these characteristics.

## Introduction

Bovine spongiform encephalopathy (BSE) is a prion disease in cattle, which is characterized by spongiform changes and accumulation of a disease-associated isoform of the prion protein (PrP^d^) in the central nervous system (1). PrP^d^, a misfolded isoform of the host cellular prion protein (PrP^C^), is the main, if not sole, component of prions (2). Initial studies indicated that BSE is a single prion strain, named classical (C-) BSE, based on the molecular characteristics of the proteinase K (PK)-resistant core of PrP^d^ (PrP^res^), neuroanatomical distribution patterns of PrP^d^ observed in most BSE cases in cattle, and C-BSE transmitted rodent models (3). In the 2000s, atypical BSEs were detected in aged cattle worldwide and were classified into at least two forms (4, 5). These two atypical forms are commonly referred to as high-type (H-BSE) and low-type (L-BSE) BSEs based on the higher or lower molecular masses of the unglycosylated forms of PrP^res^, respectively (6).

It is well-known that rodent models are very useful for studying prion diseases, i.e., for prion purification and analysis of the interference between different prion strains (7–9). L-BSE prion has been shown to be directly transmitted from cattle to hamsters (10, 11). However, previous studies have demonstrated that transmission of C- and H-BSE prions from cattle to hamsters is inefficient (11, 12), whereas mouse-passaged C-BSE prion was easily transmissible to hamsters (10). Moreover, we demonstrated successful transmission of H-BSE prion from cattle to transgenic mice overexpressing hamster PrP^C^ (TgHaNSE) (13). In a previous report, we revealed that the neuroanatomical distribution patterns of immunolabeled PrP^d^ in the brain were different among individual BSE prion strains, even after the third passage in TgHaNSE mice. In contrast to PrP^d^ immunopathological findings, Western blotting (WB) analyses demonstrated that the molecular mass of the unglycosylated form of PrP^res^ and glycoform profiles of H-BSE prion were indistinguishable from those of the C-BSE prion in TgHaNSE mice. Therefore, to investigate the effect of amino acid sequences of host PrP^C^, host genetic factors, and host microenvironment on the molecular aspects of BSE prions after interspecies transmission, we established a transmission model of TgHaNSE-passaged H-BSE prion in hamsters in this study. Subsequently, we investigated the biological and biochemical properties of three different BSE prion strains in hamsters and compared these characteristics among hamsters, TgBoPrP, and wild-type mice.

## Materials and Methods

### Inoculation of Three BSE Prions in the Animals

Animal experiments were performed in strict accordance with the regulations in the Guide for the Care and Use of Laboratory Animals of the National Institute of Animal Health and in accordance with the Guidelines for Proper Conduct of Animal Experiments (2006) by the Science Council of Japan. Procedures involving animal subjects were approved by the Institutional Animal Care and Use Committee at the National Institute of Animal Health (approval ID: 15-031), with all possible effort made to minimize pain and discomfort of the animals. Six 3 week-old female hamsters were inoculated intracerebrally with 20 μl of 10% brain homogenate (BH) from TgHaNSE mice infected with H-BSE prion, which had been passaged thrice in TgHaNSE mice.

L-BSE prion was successfully transmitted from cattle to hamsters and TgHaNSE mice (10). Four female hamsters (3 weeks old) were inoculated intracerebrally with 20 μl of 10% BH from TgHaNSE mice infected with L-BSE prion. However, C-BSE prion was passaged once in CD-1 mice before transmission to TgHaNSE mice. Finally, TgHaNSE-passaged C-BSE prion was transmitted to hamsters and passaged thrice. The details of the transmission of C- and L-BSE prions to hamsters and TgHaNSE mice were described in previous studies (10, 13). To compare the characteristic changes of the three BSE prions after interspecies transmission, we intracerebrally inoculated 20 μL of 10% BH prepared from C-, H-, and L-BSE-affected cattle into TgBoPrP and C57BL/6 mice. Three BSE prions were passaged five times in TgBoPrP mice. On the other hand, C- and H-BSE prions were passaged thrice in C57BL/6 mice, and L-BSE prion was passaged twice in C57BL/6 mice, including a blind passage.

### Histological Analysis

The left hemisphere and selected tissues were fixed in 10% buffered formalin containing 10% methanol. Formalin-fixed tissue specimens were immersed in 98% formic acid for 60 min to reduce the infectivity; this was followed by embedding in paraffin, sectioning, and staining with hematoxylin and eosin (HE) for histological evaluation by scoring vacuolar changes in nine different areas of the brain (14).

### Immunohistochemistry

Dewaxed sections were placed on silanized glass slides, treated with 3% hydrogen peroxide at room temperature (R/T) for 10 min, incubated with 10 μg/ml of PK (Nacalai Tesque, Kyoto, Japan) in PBS containing 0.1% (v/v) Triton X-100 (Nacalai Tesque) at R/T for 10 min, and immersed in 150 mM sodium hydroxide solution at 60°C for 10 min for epitope retrieval (15). Immunohistochemical staining was performed using a tyramide signal amplification (TSA) system (PerkinElmer, Waltham, MA, USA). Sections were incubated with blocking reagent for 30 min at R/T, monoclonal antibody (mAb) 44B1 (Table 1) for 1 h at R/T (16, 17), and biotinylated anti-mouse IgG (PerkinElmer) for 30 min at R/T. Slides were then incubated with streptavidin–horseradish peroxidase (SA-HRP) for 30 min at R/T, treated with biotinyl tryamide as the amplification reagent for 5 min at R/T, and incubated again with SA-HRP for 30 min at R/T before visualization with 3,3′-diaminobenzidine tetrachloride containing 10 mM imidazole, as the chromogen. Finally, the sections were slightly counterstained with Mayer's hematoxylin.

**Table 1:** Monoclonal anti-PrP antibodies used in this study.

**Source**	**Epitope location on hamster PrP**	**Immunogen**	**Vendor**	**Dilution**
6H4	145–152	Human rec PrP	Prionics, Schlieren, Switzerland	1:5,000 (WB)
SAF84	164–169	Hamster SAF	SPI-bio, Montigny le Bretonneux, France	1:5,000 (WB) 1:24,000 (IHC)
44B1	156–232^a^	Mouse rec PrP	Dr. Horiuchi (Hokkaido University, Japan)	1:12,000 (IHC)

a *The epitope is discontinuous*.

### WB Analysis of PrP^res^

In brief, 20% BH was mixed with an equal volume of detergent buffer containing 4% (w/v) Zwittergent 3–14 (Merck Japan, Tokyo, Japan), 1% (w/v) Sarkosyl (Sigma-Aldrich, St. Louis, MO, USA), 100 mM NaCl, and 50 mM Tris–HCl (pH 7.6), and incubated for 30 min with collagenase (FUJIFILM Wako Pure Chemical, Osaka, Japan; final concentration of 500 μg/ml) at 37°C. The lysate was then incubated for 30 min with PK (Roche Applied Science, Penzberg, Germany; final concentration of 40 μg/ml) at 37°C. The digestion with PK was terminated with 2 mM 4-(2-aminoethyl) benzene sulfonyl fluoride hydrochloride (Pefabloc; Roche Applied Science). Further, deglycosylation with N-glycosidase F (PNGase F; New England Biolabs, Ipswich, MA, USA) was performed per the manufacturer's instructions. The resultant solution was mixed with a 2-butanol/methanol mixture (5:1), and PrP^res^ was precipitated by centrifugation at 20,000 × g for 10 min at R/T. The pellet was resolubilized in LDS sample loading buffer (Thermo Fisher Scientific, Waltham, MA, USA). PrP^res^-enriched samples were prepared from the spleen of hamsters according to the modified method described in our previous report (18). In brief, minced spleens (200 mg) were homogenized in 50 mM Tris–HCl (pH 7.6) containing 2% (v/v) Triton X-100, 0.5% (v/v) Sarkosyl, 100 mM NaCl, 5 mM MgCl_2_, 20 mg/ml collagenase, and 40 μg/ml DNase I (FUJIFILM Wako Pure Chemical), and then incubated at 37°C for 2 h. The homogenates were digested with 80 μg/ml PK for 1 h at 37°C and centrifuged at 68,000 × g for 30 min at 4°C. The resulting pellet was resuspended in 6.25% Sarkosyl in 10 mM Tris–HCl (pH 7.6) and centrifuged at 9,000 × g for 5 min at R/T. Sodium phosphotungstate (Na-PTA; Nacalai Tesque, final concentration of 0.3%) was added into the supernatant, and the resultant solution was incubated for 30 min at 37°C with constant rotation, followed by centrifugation at 20,000 × g for 30 min at 4°C. The pellet was resuspended in LDS sample loading buffer.

Samples were electrophoresed on NuPAGE Novex 12% Bis-Tris gels with NuPAGE MOPS-SDS running buffer in accordance with the manufacturer's instructions (Thermo Fisher Scientific). The proteins were then transferred onto Immobilon-P membranes (Merck Millipore, Burlington, MA, USA). The blotted membranes were incubated with blocking reagent for 30 min and then with the anti-PrP mAbs (Table 1) at 4°C overnight. After washing with PBS containing 0.05% (v/v) Tween 20 (PBST), the membranes were incubated with goat anti-mouse IgG-HRP (Jackson ImmunoResearch, West Grove, PA, USA) for 1 h, which was followed by washing with PBST. Signals were developed with a chemiluminescent substrate (SuperSignal; Thermo Fisher Scientific). For semi-quantification, blots were imaged using a Fluorchem system (Alpha Innotech, San Leandro, CA, USA) and analyzed using an image reader software (AlphaEaseFC; Alpha Innotech) according to the manufacturer's instructions.

### PK-Sensitivity Assay

Brain samples were incubated with various PK concentrations (50, 100, 500, and 1,000 μg/ml) at 37°C for 30 min. Subsequently, the samples were subjected to WB as described above. The PrP^res^ signals were detected with mAb 6H4 and normalized by the signal intensity of PrP^res^ digested with 50 μg/ml PK.

### Statistical Analysis

To determine statistical significance, Student's *t*-tests were applied on paired data. Differences of *P* < 0.05 were considered significant. Statistical analysis was performed using KaleidaGraph software (Synergy Software, Reading, PA, USA).

## Results

### Transmission of TgHaNSE-Passaged Atypical BSE Prions to Hamsters

All hamsters inoculated with 10% BH prepared from a TgHaNSE mouse infected with the H-BSE prion showed neurological signs such as loss of appetite, wasting, inactivity, hunching, and inability to groom. They reached the terminal stage of the disease after a survival period of 445 ± 47.6 days (mean survival days ± standard deviation). Subsequent transmission to hamsters resulted in similar clinical signs, and their survival periods showed no significant differences between passage numbers (*P* > 0.05). The survival period of hamsters inoculated with 10% BH prepared from a TgHaNSE mouse infected with the L-BSE prion was 228 ± 11.0 days. Comparatively, hamsters inoculated with 10% hamster-BH infected with the L-BSE prion developed disease after a survival period of 208 ± 15.5 days at the second passage, which showed no statistical differences between the two groups (*P* > 0.05, Table 2).

**Table 2:** Transmission results of three different BSE prions to Syrian hamsters.

**Source**	**Pass #**	**n/no^a^**	**Survival period** **(mean days ± S.D.)**
Cattle H-BSE prion	1	0/6	>600
H-BSE prion passaged in TgHaNSE	1	6/6	445 ± 47.6
	2	5/5	390 ± 31.0
Cattle C-BSE prion	1	0/5	>600^b^
Mouse-adapted C-BSE prion passaged in TgHaNSE^c^	1	10/10	350 ± 6.6^b^
	2	10/10	267 ± 19.2^b^
	3	10/10	272 ± 16.2^b^
Cattle L-BSE prion	1	3/4	577 ± 127.8^b^
	2	4/4	208 ± 15.5^b^
L-BSE prion passaged in TgHaNSE	1	4/4	228 ± 11.0

a *Number of PrP^d^ accumulated hamsters out of the total number of challenged hamsters*.

b *Data were obtained from our previous studies (10)*.

c *C-BSE prion was transmitted from cattle to CD-1 mice. Subsequently, C-BSE prion passaged in CD-1 mice was transmitted to TgHaNSE mice. The brain of a diseased TgHaNSE mouse was used as the inoculum for hamsters*.

### Biochemical Characteristics of PrP^d^ Accumulated in Hamsters Infected With Three Different BSE Prions

**Figure 1:**
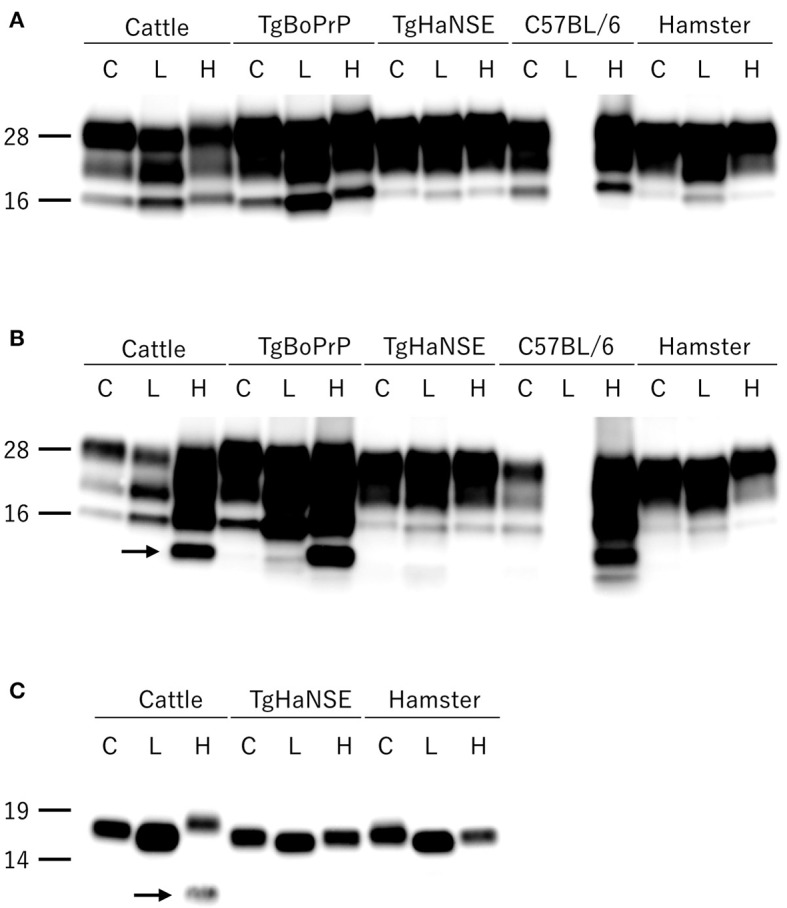
Comparison of PrP^res^ profiles among BSE strains in different host species. PrP^res^ was detected with mAb 6H4 **(A)** and SAF84 **(B)**. PrP^res^ was also detected with mAb SAF84 after PNGase deglycosylation **(C)**. PrP^res^ banding patterns, which were specific to individual BSE strains in cattle, were conserved in TgBoPrP and wild-type (C57BL/6) mice, but not in TgHaNSE mice and Syrian hamsters. Further, the L-BSE prion was not transmissible to wild-type mice. All BSE strains showed similar molecular features between TgHaNSE mice and hamsters. Arrows indicate the ~12-kDa small C-terminus PrP^res^ fragment that is specific for the H-BSE strain, but was not detected in TgHaNSE mice and hamsters infected with the H-BSE prion. C, L, and H on the top of each panel indicate C-BSE, L-BSE, and H-BSE, respectively. Molecular markers are shown to the left (kDa).

**Figure 2:**
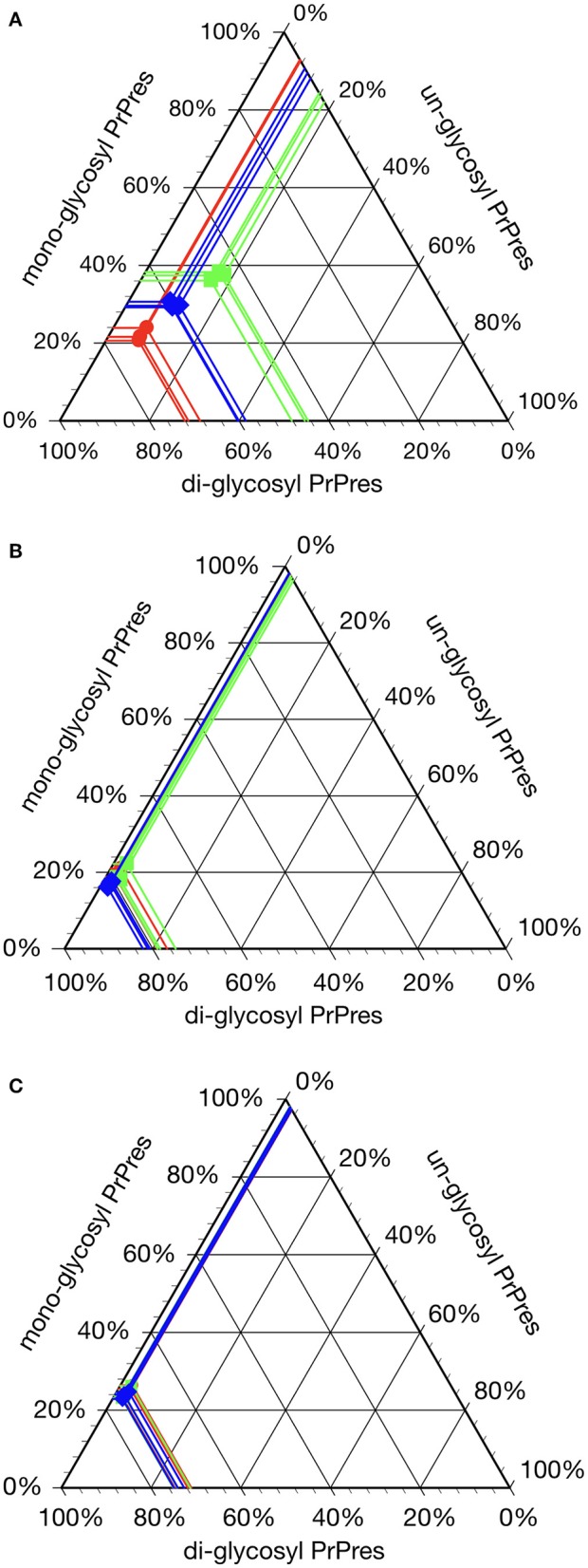
Glycoprofile analysis of C-, L-, and H-BSE strains in different host species. The relative quantity of the three major PrP^res^ bands was determined using an image reader software (AlphaEaseFC; Alpha Innotech) for glycosylation analysis (percentage of the di-, mono-, and unglycosylated PrP^res^ band). Glycoprofiles of BSE strains in cattle **(A)** were distinctly different from those in TgHaNSE mice **(B)** and hamsters **(C)**. Di-glycosylated form of PrP^res^ was dominant in all the three BSE strains in TgHaNSE mice and hamsters. Symbols: red circles, C-BSE; blue triangles, L-BSE; green squares. PrP^res^ was detected with mAb 6H4.

**Figure 3:**
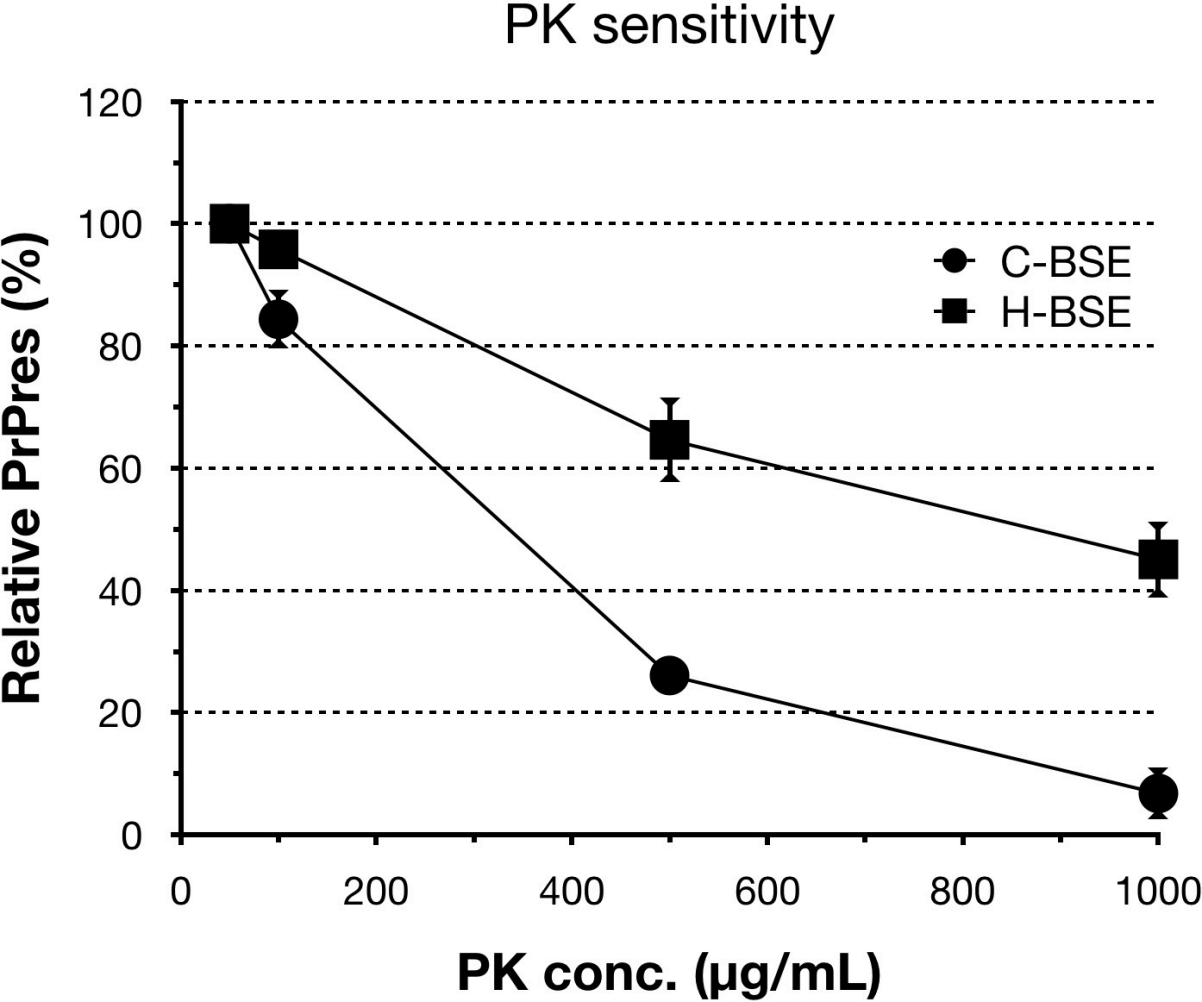
PrP^d^ sensitivity to different PK concentrations of hamster-adapted H-BSE. PK susceptibility was compared between H- and C-BSE strains in hamsters. The signal intensities were quantified by an image reader software (AlphaEaseFC; Alpha Innotech), whereby the intensity of 50 μg/ml PK concentration was set as the reference point (100%). PrP^res^ was detected with mAb 6H4. Symbols: black circles, C-BSE; black squares, H-BSE.

All hamsters infected with the H-BSE prion accumulated PrP^res^, and the major triplet PrP^res^ banding pattern detected with mAb 6H4 was identical to that in TgHaNSE mice infected with the H-BSE prion (Figure 1A). However, in WB analysis using mAb SAF84, the multiple PrP^res^ bands were not detected in TgHaNSE mice and hamsters infected with the H-BSE prion, including an additional ~12-kDa small PrP^res^ fragment, which was generally identified in cattle, TgBoPrP, and wild-type mice infected with the H-BSE prion (arrow in Figure 1B). Further, transmission of the L-BSE prion from cattle to wild-type mice did not occur, as previously reported (19). Deglycosylation of PrP^res^ with PNGase F demonstrated that the molecular mass of the unglycosylated PrP^res^ of H-BSE-infected hamsters was indistinguishable from that of C-BSE-infected hamsters and slightly higher than that of L-BSE-infected hamsters (Figure 1C). Furthermore, deglycosylation experiments confirmed the lack of the ~12-kDa small PrP^res^ fragment in hamsters infected with the H-BSE prion (arrow in Figure 1C). Three BSE prion strains showed individual glycoform profiles in cattle (Figure 2A). Conversely, glycoprofiles were identical among BSE prion strains in TgHaNSE mice and hamsters, in which the diglycosylated form of PrP^res^ was dominant (Figures 2B,C). Because the molecular mass of the unglycosylated PrP^res^ and the glycoprofiles were identical between C-BSE- and H-BSE-infected hamsters, the relative PK resistance of their PrP^d^ was compared. In contrast to C-and H-BSE prions in cattle, PrP^d^ of hamster H-BSE showed higher resistance to PK digestion than that of hamster C-BSE (Figure 3).

### Histopathology and Distribution of PrP^d^ Deposition in Brains of H-BSE-Infected Hamsters

**Figure 4:**
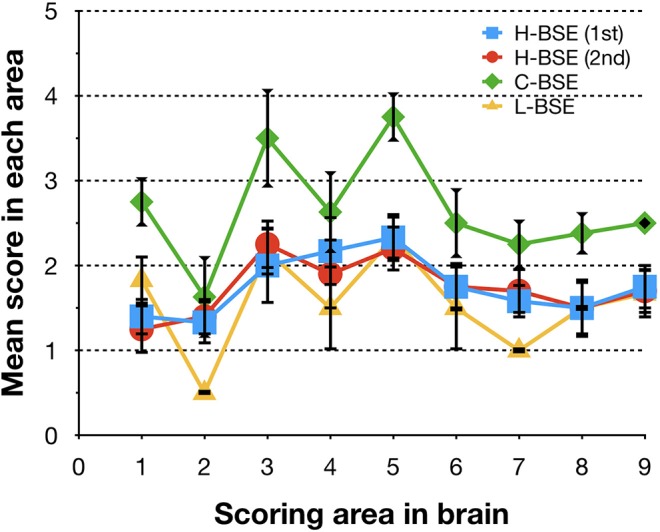
Comparison of lesion profiles of hamsters infected with three different BSE strains. Lesion profiles of hamsters inoculated with H-BSE prion passaged in TgHaNSE mice (first passage, blue square; second passage, red circle), Mouse-adapted C-BSE prion passaged in TgHaNSE mice (green diamond), and L-BSE prion from cattle (yellow triangle). Vacuolation was scored on a scale of 0–5 in the following brain areas: 1, dorsal medulla; 2, cerebellum; 3, midbrain; 4, hypothalamus; 5, thalamus; 6, hippocampus; 7, septal nuclei of the paraterminal body; 8 caudal cerebral cortex; and 9, rostral cerebral cortex. Data represent mean ± standard deviation (*n* = 4–6). The vacuolar lesion scores for C-BSE and L-BSE were taken from a previous study (10).

**Figure 5:**
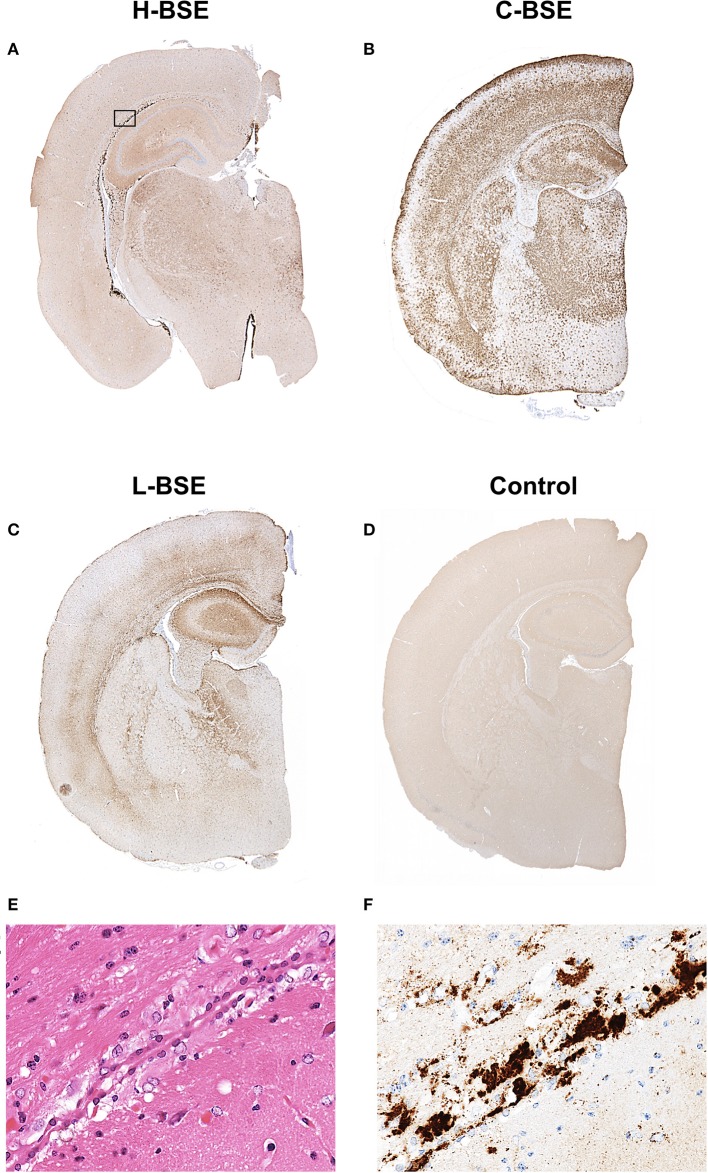
Comparison of PrP^d^ distribution in the brains of hamsters infected with three different BSE strains. Representative PrP^d^ distribution in a coronal brain slice (section) at the thalamic level of a hamster infected with H-, C-, and L-BSE is shown **(A–C)**. A brain section from a hamster inoculated with PBS is shown as a negative control for PrP^d^ immunohistochemistry **(D)**. Dense plaque-like deposits and coalescing PrP^d^ aggregates were mainly distributed in the periventricular and subcallosal regions of the H-BSE-infected hamster, but granular PrP^d^ was deposited throughout the brain of the C-BSE- and L-BSE-infected hamsters. The area within the box is shown enlarged **(E,F)**. Immunohistochemistry was performed with mAb 44B1.

Spongiform changes, represented by vacuolar lesion profiles in the brain, at both first and second passage in H-BSE-infected hamsters were very similar, and the lesion profiles were clearly different from those of hamsters infected with C- and L-BSE prions (Figure 4). The extent of vacuolation was mild to moderate throughout the brains of H-BSE-infected hamsters. Furthermore, PrP^d^ distribution patterns in the brains of H-BSE-infected hamsters were distinguishable from those in the brains of hamsters infected with C- and L-BSE prions (Figures 5A–C). No specific background staining was detected in the brain of hamsters intracranially inoculated with PBS (Figure 5D). In H-BSE prion infection, the most frequent and prominent deposits of PrP^d^ were coalescing or plaque-like deposits in the periventricular, subependymal, and subpial regions of the brain, which were not detected in C- and L-BSE-infected hamsters (Figures 5A,E,F). However, fine granular PrP^d^ was also present in the neuropil of the gray matter of the brain (Figure 5F).

### Extracerebral PrP^d^ Distributions in Hamsters Infected With H-BSE Prion

**Figure 6:**
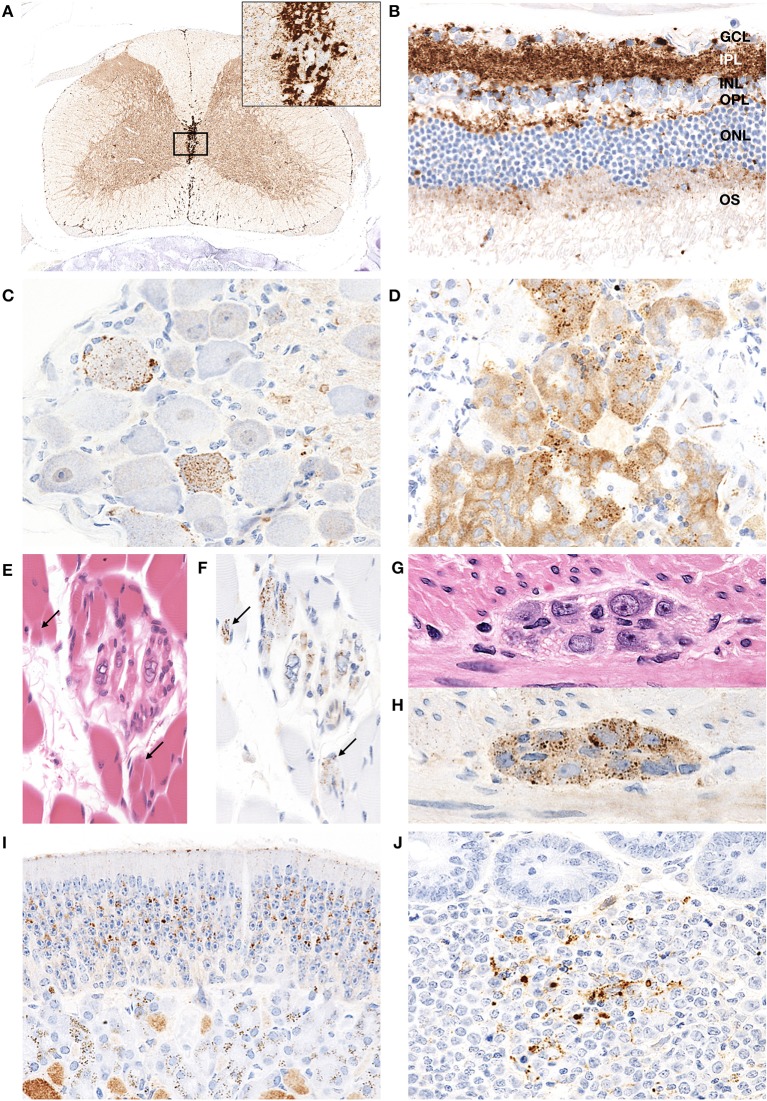
PrP^d^ distribution in extracerebral tissues of H-BSE-infected hamsters. Dense PrP^d^ aggregates are observed in the peri-central canal and subpial regions, and granular PrP^d^ deposits are conspicuous in the gray matter **(A)**. The area within the box is shown enlarged in the inset in the upper right corner. Diffuse granular deposition of PrP^d^ is observed in all layers of the retina **(B)**. GCL, ganglion cell layer; INL, inner nucleus layer; IPL, inner plexiform layer; NFL, nerve fiber layer; ONL, outer nucleus layer; OPL, outer plexiform layer; OS, outer segments. Granular PrP^d^ deposits are observed in the ganglionic and satellite cells of the trigeminal ganglion **(C)**, chromaffin cells of the adrenal medulla **(D)**, intrafusal myofiber of the muscle spindle **(E,F)**, skeletal muscle fibers (**E,F**; arrows), and neuronal cells of the myenteric plexus in the colon **(G,H)**. PrP^d^ deposits are observed in the olfactory sensory epithelium of the nasal mucosa **(I)** and the Peyer's patch, presumably corresponding to follicular dendritic cells **(J)**. Immunohistochemistry was performed with mAb 44B1.

**Figure 7:**
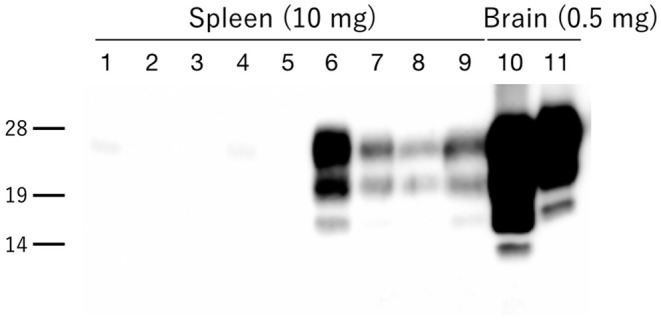
PrP^res^ accumulation in the spleens of H-BSE-infected hamsters. PrP^res^ was detected using mAb 6H4. PrP^res^ signals were detected in the spleens of four individual hamsters infected with the H-BSE prion (lanes 6–9), but not in five individual hamsters infected with the L-BSE prion (lanes 1–5). Brain samples from hamsters infected with L- and H-BSE prions were loaded as positive controls, respectively (lanes 10 and 11). The equivalent tissue quantities loaded per lane are indicated at the top, and the molecular markers are shown to the left of the panel (kDa).

Granular PrP^d^ deposits were detected in the brain and extracerebral tissues, including the spinal cord (Figure 6A), retina (Figure 6B), trigeminal ganglia (Figure 6C), adrenal medulla (Figure 6D), muscle bundles or skeletal and lingual muscle fibers (Figures 6E,F), enteric plexuses (Figures 6G,H), olfactory sensory epithelium (Figure 6I), and Peyer's patches, presumably corresponding to follicular dendritic cells (Figure 6J). In addition, WB analysis demonstrated the accumulation of PrP^res^ in the spleens of H-BSE-infected hamsters (Figure 7). The results for immunohistochemical detection of PrP^d^ in negative tissue controls are shown in Figure S1. Weak diffuse background labeling was present in the gray matter of the spinal cord (Figure S1A) and adrenal medulla (Figure S1D). Non-specific background immunolabeling was also detected in the olfactory glomeruli (Figure S1I; asterisks).

## Discussion

In this study, we demonstrate that H-BSE prion could transmit to wild-type hamsters by passaging in TgHaNSE mice prior to hamster transmission. Because no statistically significant difference was found in the survival periods between the primary and secondary passage in hamsters infected with the H-BSE prion, there is probably no strain or species barrier for H-BSE transmission between TgHaNSE mice and hamsters. Furthermore, no species barrier effect was observed in L-BSE infection from TgHaNSE mice to hamsters, indicating that the PrP^C^ amino acid sequence might primarily contribute to the adaptation of atypical BSE prions to hamsters. In contrast, survival periods have been reported to be shortened in cases of atypical BSE prion transmission from cattle to TgBoPrP mice (20, 21), suggesting that important factors involved in the adaptation of atypical BSE prions to recipients after interspecies transmission depend on the host species, although the PrP^C^ amino acid sequence would be a key factor for adaptation of atypical BSE prions.

The biological properties, such as survival periods, lesion profiles, and neuroanatomical distribution patterns, of PrP^d^ in hamsters infected with the H-BSE prion were distinct from those of hamsters infected with C- and L-BSE prions. The most characteristic finding in H-BSE-infected hamsters was the presence of plaque-like deposits, which were absent in TgHaNSE mice infected with the H-BSE prion (13). By contrast, plaque-like deposits were present in TgHaNSE mice infected with the L-BSE prion, but not in L-BSE-infected hamsters (10, 13). Therefore, differences in their PrP^C^ expression level and brain distributions may underlie these differences between transgenic mice and wild-type hamsters.

Interestingly, the biochemical properties of PrP^d^ in the brains of H-BSE-infected hamsters, including PrP^res^ banding patterns and the molecular mass of unglycosylated PrP^res^, were distinct from those of cattle and TgBoPrP mice infected with the H-BSE prion (20, 22). In addition, our data demonstrate that PK susceptibility of the three BSE prions was remarkably different between cattle and hamsters (10, 23). These results indicate that the PrP^d^ structure of each BSE prion could undergo modification from the original structure in cattle to that in hamsters through interspecies transmission. Furthermore, this change in the structure of PrP^d^ in hamsters may explain the change of the glycoprofiles in L-BSE infection between cattle and hamsters. PrP^C^ is post-translationally modified with two terminally sialylated *N*-linked glycans (24). Katorcha et al. proposed that one prion strain with minor constraints between glycans belonging to neighboring PrP^d^ monomers could recruit diglycosylated PrP^C^ for replication; however, another prion strain with substantial spatial constraints between glycans from neighboring PrP^d^ monomers would limit the percentage of diglycosylated PrP^C^ due to high electrostatic repulsion between sialic acids from neighboring *N*-glycans (25). L-BSE prion showed the strongest exclusion of the diglycosylated form of PrP^C^ in cattle (6, 26, 27), but L-BSE prion in hamsters favored the diglycosylated form for replication (Figures 1, 2), indicating that the L-BSE prion-specific alignment of glycan moieties of PrP^d^ might change after interspecies transmission to hamsters, which may allow the L-BSE prion in hamsters to efficiently recruit diglycosylated PrP^C^ molecules for its replication. Although glycoprofiles have sometimes been used for prion strain typing (28), our results demonstrated that the glycoform ratio of atypical BSE prions, like other properties (19), changed after interspecies transmission. Therefore, it does not necessarily reflect the characteristics of the original BSE prion strains.

Despite intracerebral inoculation, PrP^d^ was present in the lymphoid tissues of H-BSE-infected hamsters, in contrast to the absence of PrP^d^ lymphotropism in cattle (29, 30) and wild-type mice (31) infected with the H-BSE prion. However, PrP^res^ was not detected in the lymphoid tissues of intracranially inoculated L-BSE hamsters, as previously reported (32). Although the mechanism for lymphotropism in hamsters is unclear, there is a possibility that not only the prion strain-specific properties but also host-species genetic factors and the host microenvironment might contribute to the lymphotropism of atypical BSE prions. In future studies, PrP^d^ identified in the lymphoid tissues should be biologically characterized for a better understanding of the pathogenesis of H-BSE prion infection after interspecies transmission to hamsters.

In summary, we successfully established an H-BSE hamster model and compared the properties of PrP^d^ among H-, C-, and L-BSE prions in hamsters. Each BSE prion strain could be distinguished by its individual pathological features, including the patterns of spongiform changes and neuroanatomical distribution of PrP^d^ in the brain of affected hamsters. Furthermore, our datasets suggest that some molecular characteristics of atypical BSE prions are modified because of the influence of interspecies transmission from cattle to hamsters, resulting in some indistinguishable molecular characteristics of PrP^d^ compared to C-BSE prion in hamsters. These hamster models can be useful for further research on the pathology of BSE, a disease that poses significant challenges to the cattle industry.

## Data Availability Statement

All datasets generated for this study are included in the article/Supplementary Material.

## Ethics Statement

The animal study was reviewed and approved by The Institutional Animal Care and Use Committee at the National Institute of Animal Health. Written informed consent was obtained from the owners for the participation of their animals in this study.

## Author Contributions

KMi, KMa, and HO conceived and designed the experiments. KMi, KMa, HO, and YM performed the experiments. All the authors analyzed the data. KMi and HO wrote the manuscript. All the authors have read and approved the manuscript.

### Conflict of Interest

The authors declare that the research was conducted in the absence of any commercial or financial relationships that could be construed as a potential conflict of interest.
