# Supplementary material for: Influence of Interspecies Transmission of Atypical Bovine Spongiform Encephalopathy Prions to Hamsters on Prion Characteristics
Source: Front Vet Sci. 2020 Mar 3;7:94. doi: 10.3389/fvets.2020.00094 (PMC7062703; doi:10.3389/fvets.2020.00094)
Supplement: Supplementary file 1 [file Image_1.pdf]

Supplementary Material

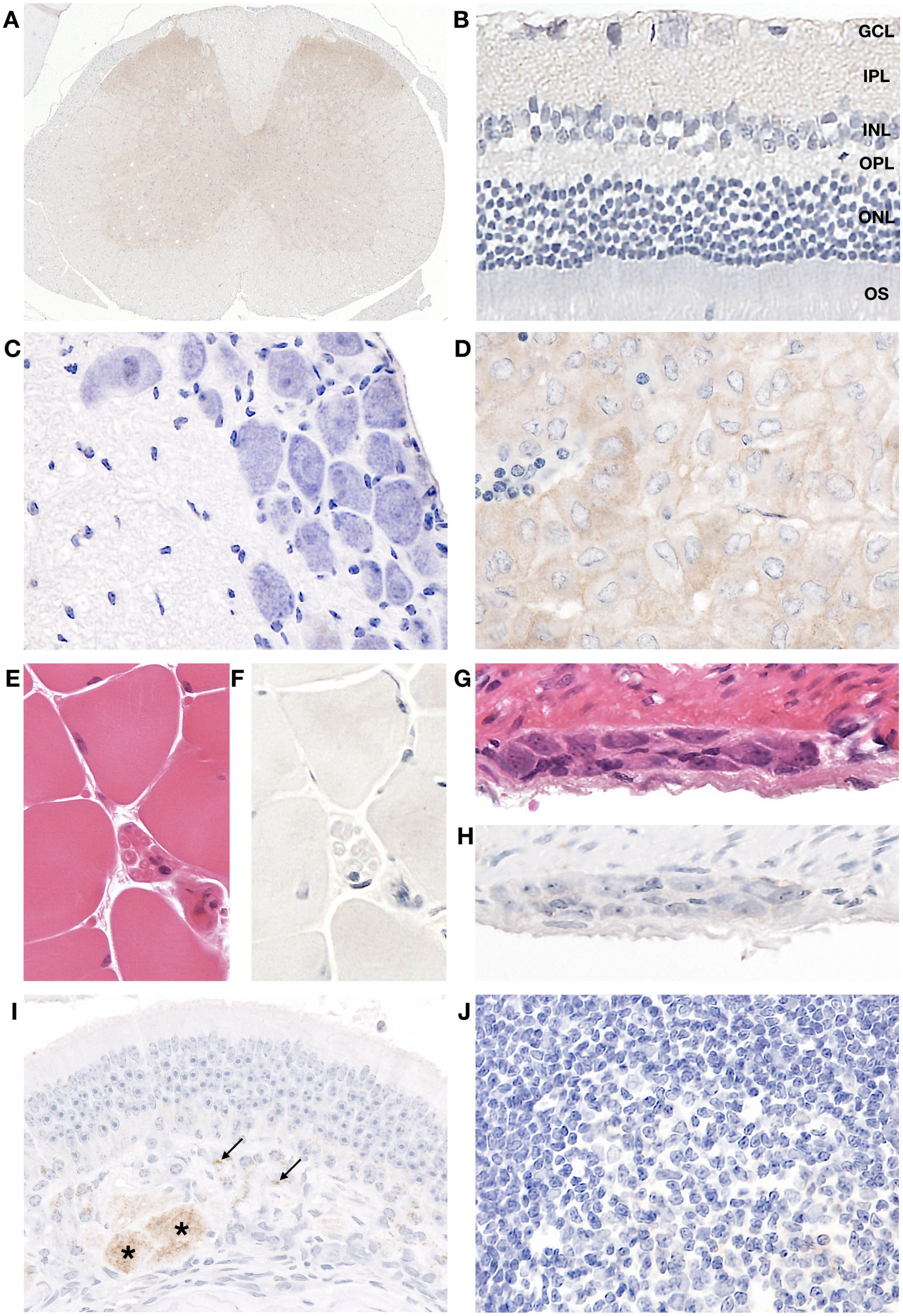

Figure S1. Immunohistochemical detection of PrP^d in extracerebral tissues used as negative controls. Spinal cord (**A**), retina (**B**), trigeminal ganglion (**C**), adrenal medulla (**D**), muscle spindle (**E and F**), myenteric plexus of the ileum (**G and H**), nasal mucosa (**I**), and Peyer's patch of the ileum (**J**). Diffused background labeling is present in the gray matter of the spinal cord, especially in the dorsal horn (**A**) and adrenal medulla (**D**). Non-specific immunolabeling is also present in the olfactory glomeruli (**I**; asterisks). In addition, brown pigments, e.g., hemosiderin or lipofuscin, can be visualized in the lamina propria of the nasal mucosa (**I**; arrows). Immunohistochemistry was performed with mAb 44B1.
